# Studying Human Fertility

**DOI:** 10.1289/ehp.112-1247503

**Published:** 2004-08

**Authors:** Michael Joffe, Jane Key, Nicky Best, Tina Kold Jensen

**Affiliations:** Department of Epidemiology & Public Health, Imperial College Faculty of Medicine, London, United Kingdom, E-mail: m.joffe@imperial.ac.uk; Institute of Public Health, Department of Environmental Medicine, University of Southern Denmark, Odense, Denmark

We very much welcome the National Children’s Study, which promises to raise the study of factors affecting reproduction and development to a new level. An impressive and exciting range of new methodologies is being developed (Chapin and Buck 2004; National Children’s Study 2004).

However, we think it important to correct some of the inaccurate statements concerning the use of retrospective time to pregnancy (TTP) made by Tingen et al. (2004). We do not see prospective methods and the retrospective approach as alternatives; they are complementary, each having their strengths and weaknesses. Unfortunately, Tingen et al. presented a negative and distorted view of retrospective TTP studies, describing things that are “often” or “typically” done but that do not represent current best practice; then they used their description to denigrate all such studies. Although it is true that retrospective studies are subject to multiple potential “bias in recruitment, recall, and behavior or exposure trends” (Tingen et al. 2004), careful sampling and questionnaire design and use of appropriate methods of analysis can address most of these issues.

Retrospective studies are not necessarily pregnancy based. They can be conducted in random population-based samples and frequently are cross-sectional or birth cohort studies (Joffe 2000; Joffe and Li 1994; Karmaus et al. 1999; Sallmén et al. 1995; Schaumburg and Boldsen 1992; Schaumburg and Olsen 1989; Thonneau et al. 1999), thereby overcoming the problem that only women who eventually conceived are included. Even in pregnancy-based studies, if there are concerns about differential prenatal care (an issue in the United States but not in western Europe, for example), recruitment could be based on births rather than pregnancies, obviously with loss of nonbirth outcomes. If sampling is population based, it is feasible to ascertain periods of unprotected intercourse not leading to conception (generally stipulating a minimum duration such as 6 months); these attempts can be added to the pregnancy-related TTP values to generate the “time of unprotected intercourse” (Karmaus et al. 1999).

Tingen et al. (2004) presented simple issues of questionnaire design negatively, but these problems can be easily solved. For example, if data are collected in relation to the starting time instead of the conception time (Weinberg et al. 1994), behavior change does not lead to bias but only to nondifferential loss of information.

A central issue is planning bias, the question being how to exclude accidental (unplanned) pregnancies without bias occurring if the exposure variable is associated with the degree of “plannedness.” Retrospective studies can readily investigate this by following the standard guidance to collect full information for all pregnancies, including all covariates, and carry out parallel analyses with “unplanned pregnancy rate” as outcome variable (Weinberg et al. 1994). Prospective studies are unable to do this because only planners are recruited.

Tingen et al. (2004) stated that in TTP studies, “women are asked to recount their contraceptive and sexual history.” This is incorrect; in TTP studies, women are not asked for this detailed information because it would be invasive and inaccurate. Instead, women are simply asked how long it took to conceive, a question that is acceptable and that most can answer. The replies give an accurate representation of the true TTP distribution (Baird et al. 1991; Joffe et al. 1993, 1995; Zielhuis et al. 1992), even with recall of up to 20 years (Joffe et al. 1995). Although digit preference (and other non-differential misclassification) can occur, the implication is that more respondents are required than would be the case with perfect information. Nevertheless, stable estimates of the TTP distribution can be obtained with approximately 200 values in each exposure group, or fewer in the case of ordered categories such as successive 5-year periods (Joffe 2000).

We agree that a major limitation of retrospective studies is that it is impossible to obtain detailed, timed information on exposures and key biologic events such as ovulation, and difficult to ascertain certain covariates such as frequency or timing of intercourse. This is the key strength of the prospective design. On the other hand, retrospective studies are representative because, as already noted, sampling from the general population is available and planning bias can be handled. The questions are easily administered and answered, and the response rate is high. Even response bias can be avoided by nesting the TTP questions within a more general population survey, thus decoupling survey nonresponse from differential fertility or other motivation that would convert low response rates to response bias (Joffe 2000). Selection bias remains a potential problem for some retrospective designs but can be handled by appropriate statistical analysis allowing for truncation effects (Scheike and Jensen 1997).

Not only are prospective studies time-consuming and costly, and therefore likely to be rarely used, but they have important methodologic drawbacks. For example, it is impossible to distinguish the approximately 3% of couples who are sterile from those who merely take a long time to conceive (> 10% typically take > 12 months), unless follow-up is extremely long.

More seriously, prospective studies are dominated by the lack of a sampling frame (except in occupational studies) and by a potent combination of planning bias and response bias. They can include only couples who deliberately plan and are willing to volunteer for onerous monitoring. This is acceptable for internal comparisons (e.g., studying day-specific conception rates, each subject being her own control) but raises serious problems with external validity. Tingen et al. (2004) referred to this only in their Table 1—“Participants might be less representative of target population”—but not in the text; in contrast, Buck et al. (2004) admitted that women who plan their pregnancies may be systematically different from those who do not, that this may adversely affect external validity to a degree which cannot be empirically evaluated, and that the findings may not be generalizable to all women.
